# Ventilatory Efficiency and End-Tidal CO_2_ Kinetics During Active Recovery Following VT_2_—Referenced Intermittent Exercise in Basketball

**DOI:** 10.3390/medicina62030552

**Published:** 2026-03-16

**Authors:** Ștefan Adrian Martin, Barbara Cintia Sándor, George Mihăță Gavra, Gabriela Szabo, Roxana Maria Martin-Hadmaș

**Affiliations:** 1 Center for Advanced Medical and Pharmaceutical Research, ”George Emil Palade” University of Medicine, Pharmacy, Science and Technology of Târgu Mures, Gheorghe Marinescu 38, 540139 Târgu Mures, Romania; 2 Department of Community Nutrition and Food Safety, ”George Emil Palade” University of Medicine, Pharmacy, Science and Technology of Târgu Mures, Gheorghe Marinescu 38, 540139 Târgu Mures, Romania

**Keywords:** basketball, cardiopulmonary exercise testing, active recovery kinetics, recovery-slope metrics, anaerobic thresold

## Abstract

*Backround and Objectives*: Basketball performance is shaped by repeated high-intensity actions interspersed with brief recovery. Conventional continuous or strictly incremental testing may not fully capture short active-recovery dynamics relevant to stop-and-go sports. *Material and Methods*: This study applied a VT_2_-referenced progressive–intermittent treadmill protocol and focused on 60-s active-recovery kinetics to describe effort tolerance in an applied basketball setting. Basketball players from Mureș County completed anthropometry (24 h pre-test, fasted) and a single laboratory visit. Pre-test training and diet were standardized for 48 h (submaximal training; predominantly carbohydrate intake). CPET was performed in 3-min stages (6.5 km·h^−1^ start; +0.7 km·h^−1^ per stage) and stopped at RER = 1.00 and/or blood lactate = 4.0 mmol·L^−1^ (operational VT2). After 3 min active recovery, participants completed six 60-s high-speed bouts separated by 60-s active recovery intervals (AR1–AR6), with intensities prescribed at 120–180% of VT2-derived speed, followed by an 8-min active recovery. For each AR interval, linear regression over 0–60 s yielded slopes for VO_2_, VO_2_/HR, VCO_2_, V̇E, VE/VO_2_, VE/VCO_2_, and PetCO_2_. *Results*: VT_1_ was determined at 2.29 m·s^−1^ (VO_2_ 32 mL·min^−1^·kg^−1^) and VT2 at 3.07 m·s^−1^ (VO_2_ 42 mL·min^−1^·kg^−1^). Maximal intermittent speed was 5.33 m·s^−1^ (VO_2_ 45.5 mL·min^−1^·kg^−1^; RER 1.06; PetCO_2_ 38 mmHg). VO_2_ differed across successive bouts (*p* = 0.0001), while PetCO_2_ showed a small downward drift across repetitions. Peak indices (max speed, VE/VCO_2_max, PetCO_2_max, VEmax) were associated with phase-specific recovery slopes across early, mid, and late recovery periods (false discovery rate–adjusted correlations). Lactate decreased over 8 min, but lactate change rates were not associated with peak indices. *Conclusions*: The VT_2_-referenced progressive–intermittent protocol appears feasible in basketball players and provides phase-dependent recovery information that complements conventional peak CPET outcomes, with potential relevance for applied team settings.

## 1. Introduction

In team sports, improving training quality and informing selection decisions increasingly rely on objective assessment strategies. Physical requirements vary by playing position, tactical role, individual potential, and training priorities, which typically reflect both competitive level and team objectives [1]. In basketball, this variability is reflected not only in anthropometric profiles (e.g., guards are typically lighter than forwards and centers), but also in key performance qualities such as lower-limb power and linear speed, alongside widely reported strength benchmarks in professional players [2,3]. Athletes often require individualized preparation to tolerate team-training demands [4]. However, differences in training structure across competitive levels are not well characterized, and their longer-term physiological and performance-related implications remain understudied [5].

Basketball is a high-intensity, intermittent team sport characterized by repeated accelerations, rapid changes of direction, jumping actions, and brief recovery intervals [6,7]. Accordingly, performance is thought to depend on the integrated contribution of aerobic and anaerobic energy systems, neuromuscular power, and movement efficiency [8]. Match analyses have reported total distances of approximately 5–6 km per game in elite players, with sustained internal physiological strain during live playing time (typically 87–95% HRmax, often >85% HRmax) and moderate-to-high lactate accumulation (commonly 3.7–13.2 mmol·L^−1^; with mean values around 5–6 mmol·L^−1^ reported in elite youth cohorts), underscoring the considerable metabolic and cardiorespiratory demands of competition [9]. Quantifying internal physiological responses in basketball presents methodological and practical challenges due to the intermittent and non–steady-state nature of play, frequent transitions between movement intensities, and the variability of locomotor patterns [10,11]. Moreover, traditional laboratory protocols often rely on continuous, steady-state exercise conditions that do not reflect the stop-and-go physiological stress experienced during competition [12,13]. To address these constraints, intermittent exercise models anchored to individualized physiological thresholds provide a controlled approach for examining cardiopulmonary and ventilatory responses under conditions that better resemble basketball-specific demands.

Aerobic capacity is considered important for sustaining performance and facilitating recovery between repeated high-intensity actions in basketball; accordingly, elite male players typically present VO_2_max values around 55–60 mL·kg^−1^·min^−1^ [14]. Moreover, faster oxygen uptake recovery kinetics have been associated with superior repeated-sprint ability, highlighting the relevance of quantifying short-window recovery behavior in stop-and-go exercise [15]. Basketball performance also relies on anaerobic power for explosive sprints and jumps, as well as the capacity to repeatedly execute short, high-intensity efforts with incomplete recovery (i.e., repeated-sprint ability, RSA) [16], which is repeatedly observed during competition in elite youth players [17]. Although laboratory- and field-based measures (e.g., VO_2_max, repeated-sprint protocols, vertical jump tests) [18,19,20] provide objective indicators of physical capacity, basketball match-play evidence shows that performance is expressed through repeated short high-intensity actions interspersed with brief recovery opportunities, resulting in rapidly fluctuating internal demands [21,22]. Consequently, these continuous, strictly incremental, or isolated assessments may not fully capture the progressive accumulation of strain and the short active-recovery dynamics that shape repeated high-intensity performance in basketball. These limitations support the need for intermittent assessment models that combine individualized intensity prescription with explicit quantification of recovery behavior, as suggested by previous research [23,24]. In the present context, this recovery behavior is summarized using ‘recovery-slope metrics’, i.e., indices describing how rapidly key physiological signals normalize during the brief active-recovery periods between successive bouts (VO_2_, VE/VO_2_, VE/VCO_2_, and PetCO_2_).

Linking standardized physiological testing with match-derived performance indicators (e.g., points, rebounds, minutes played, shooting efficiency) could improve the interpretation of physical capacities in relation to technical and tactical output [25,26]; however, relatively few studies have reported integrated physiological–match-performance datasets in basketball. To address the need for more game-representative assessment models, the present study employed a VT_2_-referenced progressive–intermittent running protocol and quantified cardiopulmonary and ventilatory responses, with particular emphasis on 60-s active-recovery dynamics, reflecting the brief recovery opportunities repeatedly described in basketball match-play time-motion and physiological analyses. Given the intermittent structure of the protocol, we hypothesized that recovery-slope metrics may offer a complementary characterization of individual effort tolerance relative to traditional cardiopulmonary exercise testing (CPET) outcomes; accordingly, the aim was to examine whether these recovery-slope metrics provide additional, phase-dependent information beyond conventional peak CPET indices.

## 2. Materials and Methods

The present study employed a cross-sectional experimental design to examine cardiopulmonary and ventilatory responses during a single VT_2_-referenced, progressive–intermittent testing session, with particular emphasis on 60-s active-recovery dynamics. All testing sessions were conducted at the Center for Advanced Medical and Pharmaceutical Research, part of ”George Emil Palade” University of Medicine, Pharmacy, Science, and Technology of Târgu Mureș, Romania. The study was approved by the University Ethics Committee (approval no. 3332 from 19 August 2024). All participants (or their legal guardians, where applicable) provided written informed consent before participation.

### 2.1. Study Sample

Participants were recruited through announcements distributed at sports clubs in Mureș County, Romania. Interested athletes underwent an initial screening consisting of health-related questions and a brief physical examination.

The study included 34 male basketball players, all actively competing in the first and second divisions of the Romanian National Basketball Championship. Eligible participants were performance-level basketball players aged 16–26 years, registered with clubs and actively involved in official competitions. Inclusion criteria required athletes to be clinically healthy, without acute or chronic medical conditions, and not undergoing any medical treatment that could influence exercise capacity. The individuals were excluded if they had cardiovascular, metabolic, or musculoskeletal conditions, were in post-injury recovery, or failed to comply with the study requirements regarding physical readiness and lifestyle habits before the test (*n* = 2).

### 2.2. Training Background

Participants were basketball players from Mureș County. In the 6 weeks before testing, training frequency was 8 sessions·week^−1^, mainly consisting of team-based technical–tactical practice (4 sessions·week^−1^), with limited and non-systematic resistance training (2 sessions·week^−1^) and additional aerobic conditioning (1 session·week^−1^). Participation in official matches was regular. Training history and weekly volume were collected by self-report. Testing was conducted during the preparatory period preceding the second half of the season; in some cases, assessments were performed during the competitive season, and all sessions were completed within a 4–6 week period.

### 2.3. Testing Procedures

All exercise testing was completed during a single laboratory visit. To minimize variability, participants were instructed to perform only submaximal training in the 48 h preceding testing and to follow a predominantly carbohydrate-based diet, similar to a pre-competition nutritional approach, while keeping meal timing consistent. This single-visit approach was chosen to reduce between-day variation in readiness (sleep, accumulated fatigue, and training load) and to limit disruption to athletes’ training/competition schedules. The 48 h pre-test instructions were implemented to minimize acute fatigue and to standardize nutritional conditions that can influence gas-exchange and ventilatory responses. Although a separate familiarization visit was not performed, the standardized warm-up and the initial low-speed treadmill stage (6.5 km·h^−1^, 3-min stages) provided on-site acclimation to treadmill locomotion and the breath-by-breath measurement setup before higher intensities.

The testing session began with anthropometric measurements, which were performed 24-h before the exercise testing session under standardized basal conditions (morning, fasted). Body mass was assessed using a Tanita MC-780MA-N body composition analyzer (Tanita Corporation, Tokyo, Japan), and height was measured with a standard stadiometer.

**Figure 1:**
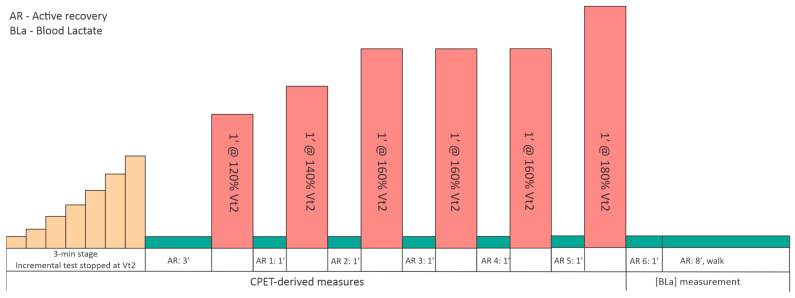
Overview of the CPET-derived testing protocol and blood lactate sampling. @ represents the number of intervals and the relative exercise intensity used in the protocol.

A standardized warm-up was completed before exercise testing, consisting of light aerobic activity and dynamic mobility exercises to prepare the cardiovascular and musculoskeletal systems for the subsequent workload. The exercise protocol comprised a stepwise incremental treadmill CPET performed in 3-min stages, consistent with established CPET methodology for ventilatory-threshold assessment and breath-by-breath interpretation [27]. Running began at 6.5 km·h^−1^ and increased by 0.7 km·h^−1^ per stage. The incremental phase was terminated when participants reached a respiratory exchange ratio (RER) of 1.00 and/or a blood lactate (BLa) concentration of 4.0 mmol·L^−1^, using a pragmatic operational criterion based on the fixed 4-mmol·L^−1^ lactate threshold concept [28]. This strategy was implemented to standardize the endpoint of the incremental phase and to avoid a fully maximal test before the subsequent intermittent bout sequence. In addition, the second ventilatory threshold (VT_2_) was identified using combined CPET criteria, including a sustained increase in ventilatory equivalents (particularly VE/VCO_2_) together with a concurrent decrease in end-tidal CO_2_ (PetCO_2_) [29]. In addition, VT_2_ was identified using combined CPET criteria, including a sustained increase in ventilatory equivalents (particularly VE/VCO_2_), together with a concurrent decrease in end-tidal CO_2_ (PetCO_2_). Immediately thereafter, participants completed 3 min of active recovery, followed by an intermittent running protocol consisting of six (n = 6) 60-s high-speed bouts interspersed with 60-s of recovery. Intermittent running intensity was prescribed relative to the VT_2_-derived running speed, with successive stages set at 120% (1 repetition), 140% (1 repetition), 160% (3 repetitions), and 180% (1 repetition) of this reference speed; each 60-s bout was separated by a 60-s active recovery (AR) interval, denoted AR1–AR6, and the protocol concluded with an 8-min active recovery, as detailed in Figure 1.

The 60-s active-recovery intervals were selected to standardize recovery assessment under stop-and-go constraints relevant to basketball match play [9,17]. This progressive–intermittent sequence was designed as a controlled treadmill analogue of basketball stop-and-go demands, using repeated short high-intensity bouts separated by brief recovery periods, consistent with established HIIT programming principles and match-play constraints in basketball [30]. Intensities were prescribed relative to the VT_2_-derived speed to individualize the suprathreshold stimulus across athletes, while the stepwise progression (120–180% of vVT2) was implemented to elicit a graded cardiopulmonary and ventilatory perturbation suitable for analyzing early (0–60 s) active-recovery kinetics [31,32,33]. Participants were closely supervised throughout the protocol. Physical condition, perceived exertion, and willingness to continue were continuously evaluated. The session concluded with a short feedback discussion and a guided static and dynamic mobility routine.

### 2.4. Cardiopulmonary Measurements and Data Acquisition

CPET data were collected breath-by-breath using a CORTEX MetaLyzer^®^ 3B-R3 (CORTEX Biophysik GmbH, Leipzig, Germany) integrated with a motor-driven treadmill set at a 0% incline (WOODWAY Pro XL; WOODWAY USA, Inc., Waukesha, WI, USA). The system was calibrated before each test using ambient air and a certified reference gas with known O_2_ and CO_2_ concentrations (16.0% O_2_ and 5.0% CO_2_) in addition to flow/volume calibration performed with a 3-L calibration syringe. Capillary blood lactate was assessed with a Lactate Pro 2 (LT-1730) analyzer (ARKRAY, Inc., Kyoto, Japan) following standardized procedures: after site disinfection (finger), the first blood drop was wiped away, and a subsequent drop was applied directly to a single-use test strip. The following physiological parameters were therefore measured and analyzed: Oxygen consumption (VO_2_, VO_2_·kg^−1^), Heart rate (HR), Ventilation (V’E), Carbon dioxide output (V’CO_2_), Respiratory exchange ratio (RER), Tidal volume (VT), Respiratory frequency (RF), Ventilatory equivalents (V’E/VO_2_, V’E/V’CO_2_), End-tidal partial pressures of O_2_ (PetO_2_) and CO_2_ (PetCO_2_), Oxygen pulse (VO_2_/HR), next to blood lactate (BLa) production.

### 2.5. Statistical Analysis

Data were analyzed using GraphPad Prism 8.0 (GraphPad Software, San Diego, CA, USA). Continuous variables are reported as median and interquartile range (median [IQR]). Data distribution was assessed using the D’Agostino–Pearson omnibus normality test. Between-bout recovery was quantified by fitting linear regressions over each 0–60 s active-recovery interval and extracting the regression coefficient (slope) for VO_2_, VO_2_/HR, VCO_2_, V̇E, VE/VO_2_, VE/VCO_2_, and PetCO_2_. Associations between peak physiological variables (e.g., maximal running speed and CPET-derived maximal indices) and recovery slopes were examined using Spearman’s rank correlation (two-tailed) and are reported as ρ and *p* values. To account for the inflation of Type I error due to multiple comparisons within the correlation matrices, the Benjamini-Hochberg procedure was applied to control the False Discovery Rate. Adjusted *p*-values (*p*_adj_) were reported, and the significance threshold was set at *p*_adj_ < 0.05.

Differences in cardiopulmonary variables across successive repetitions and across successive active-recovery intervals were tested using the Friedman test, with results reported as the Friedman statistic (Fs) and *p* values. No multiple-comparison correction was applied to these omnibus tests, as each was treated as a single global hypothesis testing the overall effect of the recovery period on a specific physiological parameter. The magnitude of effect for these omnibus tests was assessed using partial eta squared (η^2^p), interpreted as small (<0.01), medium (>0.06), or large (>0.14) based on standard thresholds. Because the present design did not include an external criterion for athlete classification (e.g., match-derived performance labels or selection status), ROC-based discrimination analyses were not applicable. In this study, sensitivity was operationalized as within-session responsiveness, defined as the ability of protocol-derived variables to detect systematic changes across successive bouts and across standardized 0–60 s active-recovery windows. Accordingly, correlation analyses were interpreted as descriptive and hypothesis-generating rather than predictive. Statistical significance was set at *p* < 0.05.

## 3. Results

The study sample had a median body weight of 86.10 kg (68.93–90.08), with 14.30% (10.90–18.30) adipose tissue and 81.29% active tissue (77.74–85.47). The total body water content was 57.80% (55.20–61.75).

### 3.1. CPET Test Results: VT_1_ and VT_2_

Within the study sample, participants reached VT_1_ at a median speed of 2.29 m·s^−1^, with an oxygen uptake of 32 mL·min^−1^·kg^−1^, 19 mL·beat^−1^ VO_2_/HR, 22.91 VE/VO_2_, 24.90 VE/VCO_2_, and 41.33 mmHg PetCO_2_. Conversely, VT_2_ was reached at 3.07 m·s^−1^, with an oxygen uptake of 42 mL·min^−1^·kg^−1^, an O_2_ pulse of 20.50 mL·beat^−1^, 26.30 VE/VO_2_, 26.13 VE/VCO_2_, and 40.16 mmHg PetCO_2_, as further described in Table 1.

### 3.2. Intermittent High-Intensity Running: Responses Across 120–180% of VT_2_ Speed

During the intermittent test, the athletes reached a median absolute speed of 5.33 m·s^−1^ at an oxygen uptake of 45.50 mL·min^−1^·kg^−1^, a VE/VO_2_ of 27.10, a VE/VCO_2_ of 27.05, an RQ of 1.06, and a PetCO_2_ of 38 mmHg.

1’ @ 120% of VT_2_ repetition was performed at 3.56 m·s^−1^. This repetition elicited an oxygen uptake of 42.54 mL·min^−1^·kg^−1^, a VE/VO_2_ of 26.16, a VE/VCO_2_ of 27.04, and 37.93 mmHg PetCO_2_, whereas the last repetition, performed at 5.33 m·s^−1^, was associated with an oxygen uptake of 47.53 mL·min^−1^·kg^−1^, an VO_2_/HR of 22.37 mL·beat^−1^, 35.33 VE/VO_2_, 32.90 VE/VCO_2_, and 38.26 mmHg PetCO_2_. Subsequently, the data for each repetition are further detailed in Table 2.

Linear trend analysis showed that proportional changes in cardiopulmonary parameters accompanied higher running speeds. Specifically, VO_2_ increased by approximately 1.04 mL·min^−1^·kg^−1^ per 1 m·s^−1^, while VO_2_/HR increased by 1.01 mL·beat^−1^ per 1 m·s^−1^. In parallel, VCO_2_ demonstrated a steeper response, increasing by 0.62 L·min^−1^ per 1 m·s^−1^, reflecting the rising metabolic and ventilatory demands at higher intensities. Further on, respiratory dynamics exhibited similar adaptations across the repetitions, as further detailed in Table 3.

Subsequently, VE/VO_2_ (*p* = 0.0001, Fs = 75.23), VE/VCO_2_ (*p* = 0.0001, Fs = 87.23), as well as VCO_2_ (*p* = 0.0001, Fs = 93.55) and PetCO_2_ (*p* = 0.0001, Fs = 63.36) were significant different across successive repetitions.

Running speed increased from 3.56 to 5.33 m·s^−1^ (Δspeed = 1.77 m·s^−1^). Over this entire speed range, median VE/VO_2_ increased from 30.65 to 35.33 (Δ = +4.68; +15.3%), VE/VCO_2_ from 29.96 to 32.90 (Δ = +2.94; +9.8%), and V̇E from 87.9 to 130.2 L·min^−1^ (Δ = +42.3; +48.1%), while PetCO_2_ decreased from 40.28 to 38.26 mmHg (Δ = −2.02; −5.0%). When expressed relative to speed increments, linear trend analysis indicated average changes of +3.39 units in VE/VO_2_, +1.83 units in VE/VCO_2_, +20.6 L·min^−1^ in V̇E, and −1.26 mmHg in PetCO_2_ per 1 m·s^−1^ increase in running speed.

### 3.3. Recovery Patterns Between Repetitions

Across the recovery period, differences in VO_2_ were recorded and confirmed by the statistical analysis (*p* = 0.0001, Fs = 57.71), similar to VCO_2_ (*p* = 0.0001, Fs = 39.55) and V̇E (*p* = 0.0001, Fs =37.37), with various afferct sezes across the studied parameters, as further detailed in Table 4.

**Figure 2:**
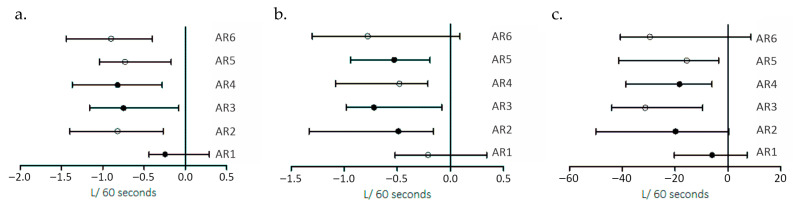
Illustrating VO_2_ (**a**), VCO_2_ (**b**), VE (**c**) recovery in the AR: 1–6 periods as median value (min to max).

Ventilatory efficiency indices generally decreased during recovery, with VE/VCO_2_ showing more pronounced reductions in between AR periods 3 and 5 (median: −3.26 in AR 3; −1.40 in AR 4; −2.44 in AR 5). Yet, the differences were not significantly different (*p* > 0.05). VE/VO_2_ declined in AR 1 and AR 3–AR 5 (median: −0.69; −1.64; −1.33; −1.63), but showed an increasing tendency in AR 2 and AR 6 (median: +1.01 and +1.90, respectively), without being significantly different over the repetitions (*p* > 0.05). In parallel, PetCO_2_ generally increased during recovery in AR 1–AR 5 (median: +0.39, +0.40, +3.74, +2.77, +1.88), whereas changes were minimal in AR 6 (median: −0.07), suggesting an attenuated end-tidal CO_2_ rebound during the final recovery period. However, throughout the recovery period, PetCO2 did not differ significantly (*p* > 0.05). Figure 2a–c. Illustrates VO_2_, VCO_2_, and VE recovery.

During the 0 to 8 min recovery period, held at the end of the test, the median lactate reduction rate was −0.63 mmol·L^−1^ per minute (−0.81 to −0.13 mmol·L^−1^). During 0–4 min, the median rate was −0.05 mmol·L^−1^ per minute (min–max: −0.38 to 1.05 mmol·L^−1^), whereas during the 4–8 min recovery period, the median rate was −1.14 mmol·L^−1^ per minute (−1.97 to −0.38 mmol·L^−1^).

### 3.4. Early Recovery (AR1–2)

During early recovery, maximal running speed was positively correlated with the VO_2_ (ρ = 0.612, *p* = 0.001, *p*_adj_ = 0.003) and VCO_2_ recovery slope (ρ = 0.646, *p* = 0.001, *p*_adj_ = 0.003). During the second recovery period, maximal speed was positively correlated with VE/VO_2_ (ρ = 0.433, *p* = 0.008, *p*_adj_ = 0.012) and V̇E (ρ = 0.437, *p* = 0.007, *p*_adj_ = 0.012) recovery slope. However, the maximum VE/VCO_2_ was significantly correlated during AR1 with both VE/VO_2_ (ρ = 0.391, *p* = 0.0241, *p*_adj_ = 0.048) and VE/VCO_2_ (ρ = 0.519, *p* = 0.002, *p*_adj_ = 0.006), alongside a negative correlation with the PetCO_2_ (ρ = −0.514, *p* = 0.002, *p*_adj_ = 0.006).

For PetCO_2_max, early recovery associations included negative correlations with VE/VCO_2_ (ρ = −0.488, *p* = 0.003, *p*_adj_ = 0.018) and the PetCO_2_ recovery slope (ρ = 0.388, *p* = 0.025, *p*_adj_ = 0.038) during AR1. During AR2, PetCO_2_max correlated positively with the VO_2_ recovery slope (ρ = 0.354, *p* = 0.034, *p*_adj_ = 0.041) and negatively with VE/VO_2_ (ρ = −0.378, *p* = 0.022, *p*_adj_ = 0.038) and VE/VCO_2_ (ρ = −0.371, *p* = 0.025, *p*_adj_ = 0.038) recovery slopes.

### 3.5. Mid Recovery (AR3–4)

During mid recovery, absolute maximal running speed showed correlations with ventilatory-efficiency data after initial statistical testing, including VE/VO_2_ (ρ = −0.412, *p* = 0.012, *p*_adj_ = 0.072) and VE/VCO_2_ recovery slopes (ρ = −0.358, *p* = 0.031, *p*_adj_ = 0.093), but indicating a non-significant trend after Benjamini-Hochberg adjustment. Regarding absolute VE/VCO_2_, significant positive correlations were observed with the VO_2_ recovery slope (ρ = 0.624, *p* < 0.0001, *p*_adj_ < 0.0001) and the VCO_2_ recovery slope (ρ = 0.410, *p* = 0.012, *p*_adj_ = 0.027) during AR3. However, during AR4, VE/VCO_2_max was negatively related to the VE/VCO_2_ recovery slope (ρ = −0.424, *p* = 0.009, *p*_adj_ = 0.027).

For PetCO_2_max, mid recovery associations included a negative correlation with the VO_2_ recovery slope (ρ = −0.480, *p* = 0.003, *p*_adj_ = 0.006) during AR3 and a negative correlation with PetCO_2_ recovery during AR4 (ρ = −0.607, *p* < 0.0001, *p*_adj_ < 0.0001). In addition, V̇Emax correlated positively with the PetCO_2_ recovery slope (ρ = 0.6434, *p* < 0.0001, *p*_adj_ < 0.0001) during AR3.

### 3.6. Late Recovery (AR5–6)

During late recovery, maximal running speed correlated negatively with the VCO_2_ (ρ = −0.530, *p* = 0.0015, *p*_adj_ = 0.009) and the VO_2_ recovery slope (ρ = −0.440, *p* = 0.0102, *p*_adj_ = 0.031). Further on, maximum VE/VCO_2_ was also significantly correlated to VCO_2_ (ρ = −0.448, *p* = 0.008, *p*_adj_ = 0.029) and PetCO_2_ recovery slopes (ρ = −0.442, *p* = 0.0098, *p*_adj_ = 0.029) during AR6. Maximum PetCO_2_ and VE/VCO_2_ recovery slope during AR5 did not reach significance after multiple testing correction (*p* = 0.0446, *p*_adj_ = 0.134), while its association with VO_2_ slope remained very large and significant (ρ = 0.634, *p* < 0.0001, *p*_adj_ < 0.0001). Consistent with this pattern, V̇E was also positively associated with the PetCO_2_ recovery slope during AR5 (ρ = 0.531, *p* = 0.0009, *p*_adj_ = 0.005). Yet, none of the peak variables showed significant correlations with lactate change rate (0–8 min, 0–4 min, or 4–8 min; all *p* > 0.05), including VE/VCO_2_max and PetCO_2_max.

## 4. Discussion

Our results indicate that this VT_2_-derived intermittent running protocol is associated with phase-dependent (early–mid–late) patterns in gas-exchange and ventilatory indices during 60-s active recovery. The recovery slopes showed several moderate-to-strong correlations with CPET-derived maximal variables, indicating that athletes with higher cardiopulmonary capacity tended to display more stable ventilatory-efficiency and CO_2_-related responses across successive recovery phases. From a practical conditioning perspective, these phase-dependent recovery profiles suggest that improving VT2-related work capacity and ventilatory efficiency may support more consistent PetCO_2_ and VE/VCO_2_ regulation during mid-to-late recovery in repeated high-intensity efforts.

### 4.1. VT_2_-Based Intermittent Prescription

The VT_2_-derived intermittent protocol proved feasible and informative in less-trained basketball players. When descriptively compared withtrained basketball players and other competitive team-sport athletes [15,16,17], the responses observed here reflect the expected physiological profile of a non-elite sample and may therefore be particularly relevant for applied team environments where conditioning status is heterogeneous [34,35,36]. In our research, the individuals reached a median absolute running speed of 5.33 m·s^−1^, accompanied by a median VO_2_ of approximately 42 mL·min^−1^·kg^−1^, a VE/VO_2_ of 27.10, and a PetCO_2_ of 38 mmHg. By comparison, competitive male basketball players are commonly reported to reach VO_2peak_ values in the range of 55–60 mL·min^−1^·kg^−1^ [37], alongside higher maximal running speeds [38] and more favorable ventilatory efficiency indices during incremental or sport-specific testing. Beyond cardiopulmonary capacity, intermittent performance in basketball is also constrained by neuromuscular qualities, including the ability to rapidly generate and effectively apply force during short ground-contact times, and to limit strength-related decrements across repeated high-intensity actions. In basketball players, repeated-sprint protocols can induce acute alterations in neuromuscular performance (e.g., force–time characteristics during jump tasks), which may contribute to variability in the mechanical cost of repeated running bouts [39]. Biomechanical evidence further indicates that running economy and sprint performance are closely linked to lower-limb muscle activation patterns and ground-reaction-force application during the support phase, highlighting that strength/power and force-application technique may interact with ventilatory and CO_2_-kinetic responses in heterogeneous, non-elite cohorts [40,41,,42]. Additionally, these data characterize repeatability during consecutive exercise bouts conducted at different intensities. Yet, as against our results, VE/VO_2_ values at comparable high intensities are often lower in trained athletes, reflecting greater ventilatory efficiency [43], while PetCO_2_ values tend to be maintained or decline less markedly at high workloads [44].

The lower VO_2_ values and the relatively elevated VE/VO_2_ observed in the present cohort suggest a reduced aerobic capacity and ventilatory efficiency, consistent with the non-competitive training background of our study sample. In this context, the VT_2_-derived velocities (3.56–5.33 m·s^−1^) likely represent a higher relative physiological strain for this group than would be expected in well-trained basketball players [34], even though the absolute speeds and gas-exchange responses remain comparatively low. These findings suggest that baseline fitness level may contribute to inter-individual differences in recovery patterns, although this interpretation cannot be definitively established without a trained comparison group. Importantly, this profile is consistent with applied team settings, particularly outside elite pathways, where squads often include athletes with diverse training histories and variable exposure to structured conditioning, which can increase inter-individual variability in physiological responses [45]. From an applied perspective, part of the variability in ventilatory efficiency and PetCO_2_ recovery may be driven by neuromuscular tolerance to repeated high-intensity actions, since fatigue-related changes in force transmission and ground-reaction-force application can elevate the metabolic cost of movement and slow recovery during typical stop-and-go sequences. Therefore, the present findings may be particularly relevant to applied contexts in which the roster does not consistently comprise the most athletic performers.

### 4.2. Phase-Dependent Recovery Behavior (Early–Mid–Late: AR1–6)

From an applied standpoint, monitoring recovery-related ventilatory responses may help practitioners identify athletes who require targeted conditioning strategies to improve repeated-effort tolerance and in-game performance sustainability. The phase-dependent recovery patterns observed in the present protocol align with previous reports showing that intermittent exercise induces time-varying cardiorespiratory kinetics across successive bouts, rather than a uniform recovery response [46]. In our less-trained sample, the absolute physiological profile was modest, based on 42 mL·min^−1^·kg^−1^ at VT_2_/upper-intensity domain and a median maximal speed of 5.33 m·s^−1^, with 27.1 VE/VO_2_ and 38 mmHg PetCO_2_, which contrasts with values frequently reported in competitive team sports athletes [47].

### 4.3. Early Recovery (AR1–2)

During early recovery, the observed associations between maximal speed and the recovery slopes of VO_2_/VCO_2_ and ventilatory indices suggest that athletes with higher maximal running speed tended to exhibit different early gas-exchange/ventilatory recovery dynamics. As to other papers, early recovery following high-intensity bouts is often characterized by a fast decline in VO_2_ and VCO_2_ and a partial normalization of ventilatory drive [16]; trained athletes typically demonstrate faster recovery kinetics and more stable ventilatory efficiency [46]. However, the comparatively modest aerobic profile (e.g., lower VO_2_ values versus competitive cohorts) may contribute to greater variability in early recovery behavior, which is consistent with the current observations [48], according to which less-trained individuals can show less consistent ventilatory control immediately after high-intensity efforts [49].

### 4.4. Mid Recovery (AR3–4)

In our paper, mid recovery was characterized by a shift in the correlation structure toward ventilatory-efficiency indices and PetCO_2_-related recovery behavior. This pattern, observed during AR3–4, suggests a redistribution of associations between maximal effort variables and recovery slopes rather than a statistically confirmed phase transition. Although direct phase-to-phase differences in ventilatory-efficiency indices and PetCO_2_ were not significant, the evolving correlation structure is compatible with prior intermittent-exercise studies showing that repeated exposures can influence ventilatory regulation and CO_2_ kinetics as cumulative strain develops [50]. In trained cohorts, ventilatory efficiency (VE/VO_2_; VE/VCO_2_) is often reported to remain more stable across repeated work [51], whereas in less-conditioned groups, greater variability may emerge, particularly under non–steady-state conditions. Given the intermittent and short-duration nature of our protocol, these mid-phase responses should therefore be interpreted as indicative of altered recovery dynamics rather than a discrete physiological shift.

### 4.5. Late Recovery (AR5–6)

In late recovery, the drop in PetCO_2_, and the pattern of significant associations involving VO_2_ and VCO_2_ recovery slopes suggest a more constrained recovery toward the end of the test. Comparable late-phase findings have been reported in repeated-bout designs, where incomplete recovery and accumulating physiological perturbations become evident in gas-exchange and end-tidal measures [52]. In the present protocol, this interpretation is supported by (i) a significant across-bout change in VO_2_ during the successive 60-s work bouts (*p* = 0.0001), (ii) a small downward drift in PetCO_2_ across repetitions, and (iii) an attenuated PetCO_2_ rebound during the final recovery interval (AR6), where the median PetCO_2_ change approached zero (−0.07 mmHg) compared with positive increases in earlier recovery periods (e.g., AR1–AR5) In this context, the relatively low baseline exercise capacity of the present sample—compared with competitive athletes—may amplify late-phase alterations and contribute to the observed phase-dependent changes. Importantly, these results should be interpreted as associations within a non-competitive cohort and may not directly extrapolate to elite squads with higher aerobic capacity and more efficient ventilatory control.

Several limitations should be considered when interpreting these findings. First, participants were less-trained basketball players with heterogeneous conditioning backgrounds; therefore, the present results primarily describe recovery behavior in this applied context and may differ in highly trained or elite cohorts with more standardized training exposure. Second, the intermittent protocol and the 60-s active-recovery windows represent non–steady-state conditions; consequently, ventilatory and gas-exchange indices (e.g., VE/VO_2_, VE/VCO_2_, PetCO_2_) may reflect transient kinetics and breathing pattern variability rather than stabilized physiological responses typically obtained during incremental CPET stages. A separate familiarization visit and test–retest reliability assessment were not conducted; therefore, potential learning effects cannot be fully excluded and should be addressed in future work. Additionally, the protocol was administered under a single condition and in a fixed sequence; therefore, order-related influences and prior-session effects could not be formally controlled. When future studies compare multiple conditions (e.g., different recovery intensities, bout durations, or prescription anchors), a counterbalanced cross-over design would help reduce potential order effects and improve internal validity. Moreover, repeating the protocol at different time points across the annual training calendar (e.g., pre-season, in-season, and post-season) would allow a more robust evaluation of the reliability and responsiveness of ventilatory efficiency and PetCO_2_ recovery metrics to changes in training status and fatigue.

The correlation analyses included a large number of tests across recovery periods and variables, increasing the risk of type I error; accordingly, these associations should be interpreted as exploratory. In addition, because no match-performance outcome or classification criterion was available in this cross-sectional design, ROC-based discrimination and multivariable predictive modelling were outside the scope of the present study and should be addressed in future work.

## 5. Conclusions

Under the present conditions, a VT_2_-referenced intermittent running framework provided a practical way to standardize intermittent work in a non-competitive basketball cohort. The recovery responses were not uniform across the session, indicating that interpreting intermittent exercise from a single “average” recovery profile may overlook meaningful within-session variation. The observed associations between recovery slopes and maximal CPET-derived indices suggest that short-window recovery metrics can complement traditional peak measures when describing intermittent exercise tolerance in applied team settings. However, given the exploratory nature of the correlation analyses and the conditioning profile of the sample, these findings should be viewed as descriptive and hypothesis-generating rather than definitive or causal.

## Figures and Tables

**Table 1:** Median CPET-Derived Variables at VT_1_ and VT_2_.

	VT_1_		VT_2_	
CPET-Deriver Variables	Median (IQR)	CV%	Median (IQR)	CV%
**Speed**, m·s^−1^	2.29 (2.19–2.73)	11.52	3.07 (2.73–3.36)	12.05
**VO_2_**, mL·min^−1^·kg^−1^,	32 (27.63–35.81)	12.82	42 (36.33–43)	11.75
**VO_2_/HR**, mL·beat^−1^	19 (15.09–20)	15.46%	20.50 (16.51–21)	12.4
**VE/VO_2_**, L·min^−1^	22.91 (22.01–25.23)	10.51	26.30 (23.48–27.23)	11.4
**VE/VCO_2_**, L·min^−1^	24.90 (23.95–26.91)	10.06	26.13 (23.78–27.27)	10.73
**VCO_2_**, L·min^−1^	2.49 (2.19–2.68)	13.48	3.11 (2.92–4.00)	15.41

**Table 2:** Descriptive data regarding CPET parameters (VO_2_, VO_2_/HR, VCO_2_) during each VT_2_-derived repetition.

MOMENT	Speed, m·s^−1^	VO_2_, mL·min^−1^·kg^−1^				VO_2_/HR, mL·beat^−1^				VCO_2_, L·min^−1^			
		Median	IQR	CV%	η^2^p	Median	IQR	CV%	η^2^p	Median	IQR	CV%	η^2^p
Bout 1: 1’ @ 120% of VT_2_	3.56	42.54	33.12–43.82	19.47	0.579	19.85	17.80–23.78	21.67	0.391	2.93	2.57–3.56	17.30	0.639
Bout 2: 1’ @ 140% of VT_2_	4.17	46.16	41.39–50.72	15.76		22.20	20.12–25.95	18.13		3.74	3.44–4.32	15.69	
Bout 3: 1’ @ 160% of VT_2_	4.75	41.75	39.90–44.63	14.16		21.31	18.16–24.00	17.33		3.60	3.00–3.94	14.61	
Bout 4: 1’ @ 160% of VT2	4.75	41.18	36.92–45.38	15.89		21.11	18.87–23.92	16.63		3.30	2.98–3.83	13.79	
Bout 5: 1’ @ 160% of VT_2_	4.75	41.75	36.73–45.18	14.36		21.21	17.87–22.30	15.93		3.415	2.95–3.72	14.56	
Bout 6: 1’ @ 180% of VT_2_	5.33	47.53	43.68–55.51	16.83		22.37	20.21–25.01	15.69		4.421	3.74–4.72	18.00	

**Table 3:** Descriptive data regarding CPET parameters (VE/VO_2_, VE/VCO_2_, VE, PetCO_2_) during each VT_2_-derived repetition.

MOMENT	Speed, m·s^−1^	VE/VO_2_				VE/VCO_2_				VE, L·min^−1^				PetCO_2_, mmHg			
		Median	IQR	CV%	η^2^p	Median	IQR	CV%	η^2^p	Median	IQR	CV%	η^2^p	Median	IQR	CV%	η^2^p
1’ @ 120% of VT_2_	3.56	30.65	28.62–35.21	10.23	0.508	29.96	28.65–32.88	8.56	0.498	87.89	73.87–111.4	24.24	0.642	40.28	39.32–43.25	9.23	0.383
1’ @ 140% of VT_2_	4.17	29.58	27.60–31.85	8.96		29.57	28.17–31.93	8.15		113.4	99.52–134.8	20.43		40.48	40.04–42.68	7.13	
1’ @ 160% of VT_2_	4.75	34.29	30.77–37.12	10.79		32.40	28.84–33.81	10.29		115.6	90.4–127.4	21.42		40.54	38.34–43.38	8.57	
1’ @ 160% of VT_2_	4.75	33.87	32.12–35.16	8.26		31.06	29.90–33.54	7.45		111.8	93.59–123.8	19.59		38.56	36.98–40.55	10.08	
1’ @ 160% of VT_2_	4.75	35.01	32.23–36.14	7.07		31.23	30.38–34.68	10.04		112.1	95.44–125.8	21.72		38.30	36.64–40.40	8.77	
1’ @ 180% of VT_2_	5.33	35.33	28.49–31.44	10.41		32.90	31.35–39.35	9.33		130.2	117.4–164.6	25.72		38.26	37.77–40.84	5.42	

**Table 4:** Effect Size Analysis Across Active-Recovery Periods (AR1–AR6).

Moment	VO_2,_ mL/min/kg			VE/VO_2_				VE/VCO_2_		VE, L/min			VCO_2_, L/min			PetCO_2_, mmHg		
	Median	IQR	η^2^p	Median	IQR	η^2^p	Median (IQR)	IQR	η^2^p	Median (IQR)	IQR	η^2^p	Median (IQR)	IQR	η^2^p	Median (IQR)	IQR	η^2^p
AR1	−0.24	−0.43 to −0.04	0.200	−0.575	−1.63 to 1.90	0.215	−0.730	−1.23 to −0.42	0.260	−5.02	−14.9 to 3.23	0.322	−0.280	−0.44 to 0.34	0.126	0.360	−0.12 to 0.75	0.20
AR2	−0.70	−0.92 to 0.03		1.25	−1.60 to 5.98		−0.235	−1.73 to 2.24		−19.9	−24.9 to 0.41		−0.555	−0.80 to −0.16		−0.205	−2.01 to 3.15	
AR3	−0.80	−0.96 to −0.08		−1.52	−7.35 to 6.48		−2.94	−4.90 to 1.60		−29.3	−41.7 to −9.53		−0.690	−0.84 to −0.08		2.02	−0.15 to 5.46	
AR4	−0.80	−0.92 to −0.28		−0.335	−1.57 to 1.70		−1.30	−3.73 to −0.58		−21.6	−29.0 to −6.02		−0.515	−0.82 to −0.21		1.85	0.21 to 5.34	
AR5	−0.79	−0.97 to −0.17		−1.18	−2.02 to 3.81		−1.51	−2.74 to 1.12		−16.1	−28.1 to −11.1		−0.530	−0.72 to 0.86		1.51	0.05 to 4.79	
AR6	−0.985	−1.34 to −0.740		2.09	−0.070 to 6.40		0.730	−0.420 to 1.96		−21.3	−34.6 to 8.68		−0.800	−1.07 to −0.660		−0.235	−2.91 to 2.22	

## Data Availability

The original contributions presented in the study are included in the article; further inquiries can be directed to the corresponding author.

## Abbreviations

The following abbreviations are used in this manuscript:

AR	Active recovery
BLa	Blood lactate
CPET	Cardiopulmonary exercise testing
HR	Heart rate
IQR	Interquartile range
PetCO_2_	End-tidal partial pressure of carbon dioxide
RER	Respiratory exchange ratio
RSA	Repeated-sprint ability
RQ	Respiratory quotient
VE	Minute ventilation
VE/VCO_2_	Ventilatory equivalent for carbon dioxide
VE/VO_2_	Ventilatory equivalent for oxygen
VO_2_	Oxygen uptake
VO_2_/HR	Oxygen pulse
VCO_2_	Carbon dioxide production
VT_1_	First ventilatory threshold
VT_2_	Second ventilatory threshold
